# The crucial role of hypertension in determining latent classes of metabolic syndrome in northern Iran and predictive power of these classes in non-alcoholic fatty liver: a gender-based insight

**DOI:** 10.3389/fendo.2025.1405833

**Published:** 2025-02-28

**Authors:** Bahareh Amirkalali, Parvin Hassanzadeh, Fatemeh Sheikholmolooki, Esmaeel Gholizadeh, Azam Doustmohammadian, Fahimeh Safarnezhad Tameshkel, Nima Motamed, Mansooreh Maadi, Masoudreza Sohrabi, Elham Sobhrakhshankhah, Farhad Zamani, Hossein Ajdarkosh

**Affiliations:** ^1^ Gastrointestinal and Liver Diseases Research Center, Iran University of Medical Sciences, Tehran, Iran; ^2^ Department of Nutrition, Health and Statistics Surveillance Research Center, Science and Research Branch, Islamic Azad University, Tehran, Iran; ^3^ Department of Social Medicine, Zanjan University of Medical Sciences, Zanjan, Iran

**Keywords:** latent class analysis, metabolic syndrome, Iran, non-alcoholic fatty liver, hypertension

## Abstract

**Introduction:**

This study investigates the subclasses of metabolic syndrome (Mets) and their relationship with non-alcoholic fatty liver (NAFLD) and the probable predictive role of serum vitamin D and CRP levels.

**Methods:**

This community-based, cross-sectional study was performed on adults in the framework of the Amol cohort prospective study (AmolCPS). Mets was defined as Adult Treatment Panel III criteria (ATP III) and ultrasound was used to diagnose NAFLD. Anthropometric and blood pressure measurements were conducted, and biochemical measurements were assessed after fasting. Data analysis included Latent class analysis, two-tailed χ2 statistics, one-way analysis of variance, and logistic regression using Mplus (version 7.4) and spss (version 26) softwares.

**Results:**

The study involved 2308 participants, with a mean age of 43.17 ± 12.30 years. Mets prevalence was 25.64%, with three identified classes: Mets with Hypertension (HTN), Mets without HTN (Non-HTN), and Low Risk. Mets with HTN had a high probability of at least four components, particularly high SBP. Non-HTN had at least three high probable components, especially high TG and low HDL but not high SBP and DBP. The low-risk class had a low probability of all components except low HDL in women. Serum vitamin D and CRP levels did not significantly predict Mets classes in men, while CRP level significantly predicted the HTN class in women (OR:1.03, CI:1.004-1.067). Both HTN, and Non-HTN Mets classes significantly increased the odds of NAFLD compared to the low risk class, especially in women (HTN class OR: 4.20 vs 2.94; non-HTN class OR: 5.60 vs 3.12 in women and men respectively).

**Conclusion:**

The latent class analysis in northern Iran identified three Mets classes: HTN, Non-HTN, and low-risk, with hypertension playing a crucial role in determining these classes. These classes were stronger predictors of NAFLD in women. Serum CRP and vitamin D levels did not emerge as significant predictors of the classes, except for serum CRP in the HTN class among women.

## Introduction

Metabolic syndrome is characterized by a cluster of interconnected metabolic abnormalities that elevate the risk of stroke, cardiovascular diseases (CVD), type 2 diabetes (T2D), non-alcoholic fatty liver disease (NAFLD), and various other health complications (1–3). The prevalence of metabolic syndrome in the global adult population is approximately 20-25% (4–6) and it continues to rise, posing a significant public health concern worldwide (1, 7). This upward trend has also been observed in Iran. In 2018, the prevalence of metabolic syndrome was estimated at 25.5% in women and 17.16% in men (8), which further increased to 34% in women and 22% in men by 2020 (9).

Metabolic syndrome has been described in various manners over the past decades, however, the metabolic abnormalities typically considered for its diagnosis consist of abdominal obesity (characterized by a large waist circumference or high waist-to-hip ratio), elevated fasting blood glucose, increased fasting serum insulin, reduced serum high-density cholesterol (HDL), elevated serum triglyceride levels (TG), and high blood pressure (10–13). In most definitions, the simultaneous presence of three or more of these components in an individual indicates the presence of metabolic syndrome, regardless of which specific three components are present. Previous research suggests that there may be distinct subgroups within this classification, with each subgroup potentially representing a different pathophysiologic state and carrying varying levels of risk for chronic diseases (14, 15).

Latent class analysis is a statistical method that enables the identification of distinct subgroups of metabolic syndrome (16) and reveals the alignment of metabolic syndrome components within a population (17). Latent class analysis utilizes a model-based approach to cluster individuals into different groups based on their responses to observed categorical variables, while also estimating latent variables from observed indicator variables (17). In Iran, several studies have utilized the Latent class analysis to investigate metabolic syndrome and its components (17–20). However, it is important to note that the prevalence of metabolic syndrome, its components, and its risk factors vary across geographical regions (21–23). Therefore, the identification of latent classes of metabolic syndrome within each specific area can greatly assist doctors, healthcare providers, and policymakers from the Health Ministry in developing tailored preventive programs for the residents of that particular region (15). Previous research has primarily focused on the relationship between latent classes of metabolic syndrome and CVD (14, 19, 24, 25) However, to the best of our knowledge, no studies have explored the association between latent classes of metabolic syndrome and NAFLD. This study also takes into consideration the role of serum vitamin D (26–29) and C-reactive protein (CRP) levels (30–32), two important factors associated with NAFLD, in determining the classification of individuals within the different metabolic syndrome subclasses. So, this study aims to investigate: 1) whether there are any subclasses of people with different profiles of metabolic syndrome components in the north of Iran, 2) whether there is a relationship between individual characteristics (such as age, BMI, serum vitamin D, and CRP level) and the placement of individuals in any of the subclasses, and 3) whether there is an association between the identified subclasses and NAFLD.

## Methods

### Study population

This community-based cross-sectional study was conducted in the framework of the Amol cohort prospective study (AmolCPS), the second phase. A detailed description of AmolCPS was explained in another article (33) but briefly, this study was conducted in Amol, a city in the northern region of Iran, in two different periods: 2009-2010 (phase 1) and 2016-2017 (phase 2). Study subjects were selected from 25 rural and 16 urban healthcare centers between the ages of 10 to 90. Then, the subjects were divided into 16 groups based on gender and age with ten-year intervals, 10-19, 20-29, 30-39, 40-49, 50-59, 60-69, 70-79, and 80-89. The selection of studies in each stratum was done using a simple randomization method, which was proportional to the population of each stratum.

The present study included all adults aged 18 to less than 65 years who had complete abdominal ultrasound and blood test results in the second phase of the study. Participants who followed a specific diet or exercise routine, had a history of liver disorders such as Wilson’s disease, autoimmune liver disease, hemochromatosis, viral infections, alcoholic fatty liver, malignancies, thyroid problems, autoimmune disorders, high alcohol consumption (more than 30 g/day in men and more than 20 g/day in women) (34, 35), were pregnant or breastfeeding were excluded from the study. Ultimately, 2308 individuals, comprising 1135 men and 1173 women, were included for analysis. The mean imputation method (replacing the missing values with the mean of the relevant variables) was used to address a total of 13 missing data points for weight and height.

### Ethical approval

This study has been approved by the ethics committee of Iran University of Medical Sciences by No. IR.IUMS.REC.1400.982 and was conducted according to the Helsinki Declaration. The participants entered the study after signing the informed consent form with full knowledge of the study objectives.

### Clinical information

In AmolCPS, medical, pharmaceutical, and demographic information was collected by a standard questionnaire after signing the informed consent form (33). Data on physical activity were collected using a validated International Physical Activity Questionnaire (IPAQ), which was expressed as metabolic equivalent minutes per minute per week (MET-min/week) (36) Anthropometric measurements (weight (kg), height (m), and waist circumference (cm)) were done based on the standard protocol (37) and Body mass index (BMI) was calculated as dividing weight in kilograms by the square of height in meters. Blood pressure was measured in a quiet room after 15 minutes of rest with a mercury sphygmomanometer. The average of 2 times of measurement with an interval of 1 minute was considered as the systolic and diastolic blood pressure of the person.

Then, a venous blood sample was taken after 12 hours of fasting for biochemical measurements (including fasting plasma glucose (FPG), serum triglycerides (TG), high-density lipoprotein (HDL), C-reactive protein (CRP), 25(OH) vitamin D, Creatinine (Cr), Hemoglobin A1C (HbA1c)). According to the protocol using the BS200 Auto analyzer (Mindray, China), FPG, TG, Cr, and HDL were assessed enzymatically. The HbA1c level was measured by a Variant machine (Bio-Rad, Hercules, CA, United States). Serum concentration of 25 (OH) vitamin D was measured using an ELISA Kit (Pishtaz Teb Zaman Diagnostics, Tehran, Iran), and serum CRP level was quantitatively measured using a Bionic CRP kit, Tehran, Iran.

### Definition of metabolic syndrome components

Each of the metabolic syndrome components was determined based on the definition of National Cholesterol Education Program Adult Treatment Panel III criteria (ATP III) as follows (38):

- FPG ≥100 mg/dl or drug treatment for elevated blood glucose
- Waist Circumference (WC) >102 cm in men or > 88 cm in women
- Serum TG ≥150 mg/dl or drug treatment for elevated triglycerides
- Serum HDL < 40 mg/dl in men or < 50 mg/dl in women or drug treatment for low HDL
- Blood Pressure ≥130/or ≥85 mm Hg or drug treatment for elevated blood pressure

### NAFLD diagnosis

Ultrasound was used to diagnose NAFLD. Sagittal, longitudinal, lateral, and intercostal views were obtained with a 3-5 MHz transducer (Esaote SpA, Genova, Italy). The normal liver was defined when the liver consistency was homogeneous, displayed fine-level echoes, and was minimally hyperechoic or even isoechoic in contrast to a regular renal cortex. Steatosis, on the other hand, was identified as a mild to severe increase in liver echogenicity, with severe cases exhibiting limited penetration of the posterior segment from the right hepatic lobe and poor or no visual images of hepatic vessels and diaphragm, in those without a history of excess alcohol consumption, drug-induced steatosis or viral and hereditary steatogenic hepatic conditions (39, 40). Ultrasound examinations were performed by a radiologist who was completely blind to the study protocol while the subjects were fasting.

### Statistical analysis

The latent class analysis was used to analyze data. In this statistical procedure, different subgroups are qualitatively identified in a population that have some visible characteristics in common [in this study, six dichotomous observable variables of metabolic syndrome including high WC, high FPG, low HDL, high TG, High SBP, and high DBP, based on specific thresholds for metabolic syndrome definition (38)]. These subgroups are called latent classes (41). In the first step, for determination of the number of classes, the model is fitted with one class, and then in the next steps, another class is added to the model in each run. With the help of indices and tests (such as Akaike’s Information Criterion (AIC) and Bayesian Information Criterion (BIC), Le Mandel Rubin’s likelihood ratio test (LMR LR test), and entropy (Entropy)), the model with the appropriate number of classes is selected. In our study, individuals were assigned to latent classes based on their highest posterior probability of membership. Specifically, we used a threshold of 0.50 for class assignment, meaning that individuals with a probability greater than or equal to 0.50 were assigned to the corresponding class. Then we employed the BIC and LMR test as primary criteria for selecting the optimal number of classes. The BIC is a widely used criterion in model selection that balances model fit with complexity, penalizing models with more parameters to avoid overfitting. A lower BIC value indicates a better-fitting model. The LMR test, on the other hand, compares a k-class model to a k-1 class model by assessing whether the added complexity of a new class significantly improves the fit of the model. A significant result (p-value < 0.05) indicates that the k-class model provides a better fit than the k-1 class model. During our analysis, we examined multiple models ranging from one to five classes. For each class, the BIC was calculated. The BICs of the classes were compared and finally the class with the lowest BIC to avoid overfitting and underfitting and significant LMR LR test p-value (p-value < 0.05) was selected as the model. We considered gender differences in WC and serum HDL cutoffs in the definition of metabolic syndrome but used the same metrics, such as BIC, AIC, entropy, and the Lo-Mendell-Rubin test, for both sexes to be consistent in our analytical approach.

The Shapiro-Wilks test was utilized to assess the normality of continuous variables, and the assumption of normality for each variable included in the parametric analyses was specifically evaluated. Two-tailed χ2 statistics were utilized to explore differences for categorical variables. One-way analysis of variance (ANOVA) and student’s t-tests were applied to assess differences in continuous variables with a normal distribution, while non-parametric Kruskall-Wallis and Mann-Whitney tests were used to investigate differences for continuous variables without normal distribution. Additionally, Bonferroni *post hoc* tests were used for pairwise comparisons in the ANOVA Test, and adjusted Bonferroni *post hoc* tests were used in the Kruskal Wallis test. The association between subclasses of metabolic syndrome and other risk factors such as age, BMI, serum CRP, and 25(OH) vitamin D were evaluated using Multinomial Logistic Regression. Finally, logistic regression was used to analyze the relationship between metabolic syndrome subclasses and NAFLD adjusted for age, diabetes diagnosis, serum vitamin D, CRP, and Cr level. The low-risk class served as the reference group in regressions. Mplus (version 7.4) and spss (version 26) software were used for data analysis.

## Results

### Participant characteristics

**Figure 1:**
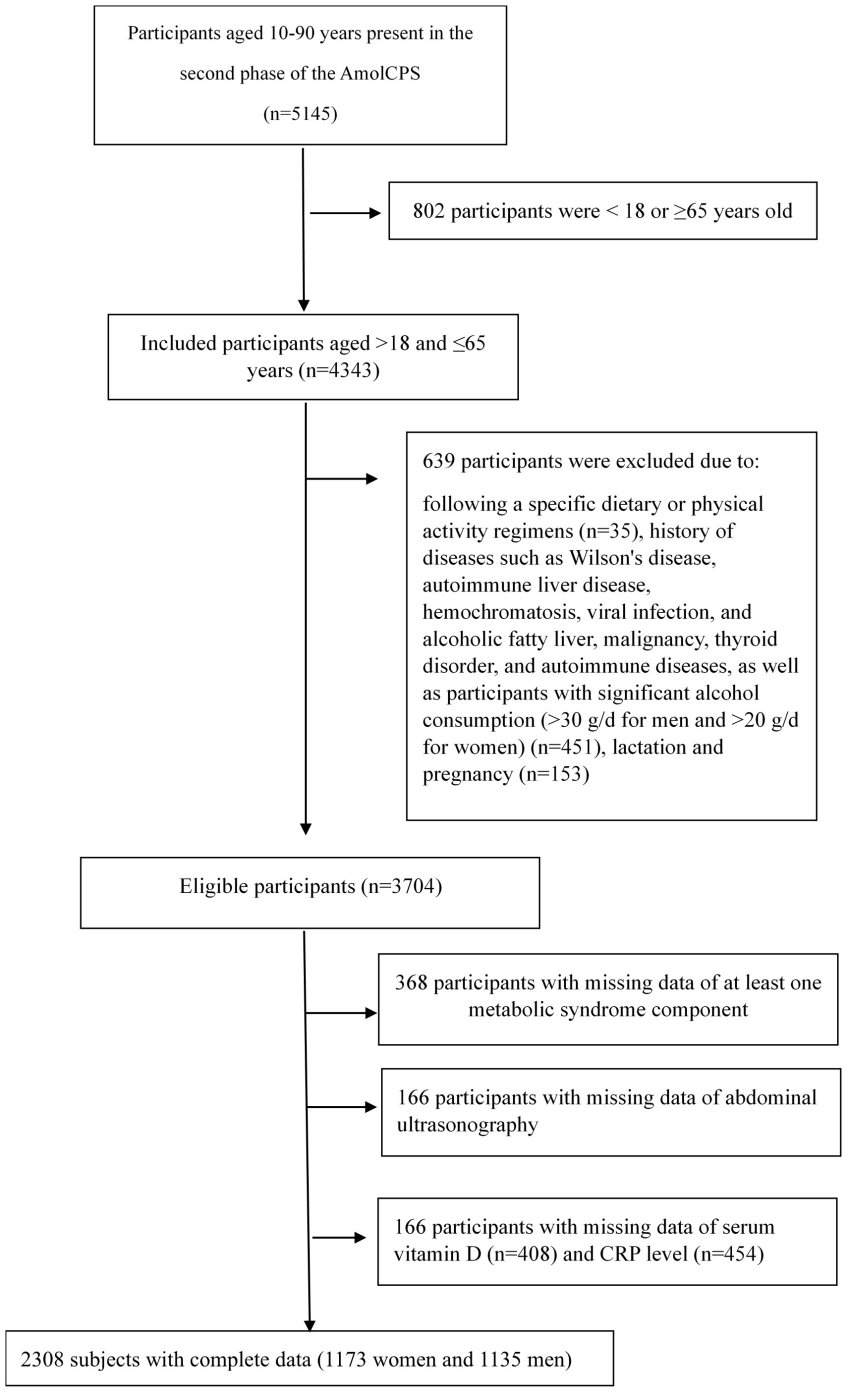
Inclusion-exclusion flow chart.

**Figure 1** displays a flowchart outlining the criteria for inclusion and exclusion. This study included 2308 participants (men=49.17%) with a mean age of 43.17± 12.30 years. According to ATPIII criteria, the prevalence of metabolic syndrome was 25.64% in the study population with a significantly higher prevalence among women (33.3% vs. 17.7%, p<0.001). Women were significantly younger (42.58 ± 12.28 vs 43.78 ± 12.30, p=0.019), with higher BMI (29.68 ± 5.74 vs 26.54 ± 4.24, p<0.001), and higher prevalence of elevated WC (46.1% vs 8%, p<0.00) and SBP (17% vs 13.9%, p=0.043). Women also had significantly higher serum CRP levels (2.00 (1.00-4.90) vs 1.00 (0.40-2.50), p<0.001) and lower serum vitamin D (14.70 (8.70-22.67) vs 19.34 (14.00-24.40), p=0.001) and Cr (0.96 (0.87-1.04) vs 1.13 (1.04-1.25), p<0.001) levels. Low serum HDL level was significantly more prevalent in men (52.4% vs 29.8%, p<0.001). They also displayed a significantly higher level of physical activity and smoking habits but had a lower rate of diabetes diagnosis. HbA1c and other components of metabolic syndrome including high serum TG, FPG, and DBP were not significantly different between the two sexes (**Table 1**).

**Table 1:** Baseline characteristic of the study participants (N =2308).

Variable	Men (n =1135)	Women (n =1173)	Total (n =2308)	P value
Age (year)	43.78 (12.30)	42.58 (12.29)	43.17 (12.31)	**0.019** ^a^*
BMI (kg/m^2^)	26.54 (4.24)	29.69 (5.74)	28.14 (5.30)	**<0.001** ^a^*
PA (MET)	4162.0 (2724.0-5437.0)	3291.0 (2252.0-4741.0)	3700.0 (2404.0-5153.5)	<0.001 ^c^*
Positive Smoking status (%)	265 (23.3)	5 (0.4)	270 (11.7)	<0.001 ^b^*
Diabetes (%)	105 (9.3)	200 (17.1)	305 (13.2)	<0.001^b^*
Serum Vitamin D (ng/mL)	19.34 (14.00-24.40)	14.70 (8.70-22.67)	17.30 (10.80-23.70)	**0.001** ^c^*
Serum CRP (mg/L)	1.00 (0.40-2.50)	2.00 (1.00-4.90)	1.40 (0.70-3.80)	**<0.001** ^c^*
HbA1c (%)	4.56 (4.00-4.80)	4.56 (4.20-4.70)	4.56 (4.10-4.70)	0.12
Serum Cr (mg/dl)	1.13 (1.04-1.25)	0.96 (0.87-1.04)	1.04 (0.93-1.16)	**<0.001** ^c^*
WC (cm)	88.58 (10.32)	87.61 (12.43)	88.09 (11.45)	**0.043** ^a^*
SBP (mm Hg)	110.00 (102.50-120.00)	109.00 (99.00-120.00)	110.00 (100.00-120.00)	**0.033** ^c^*
DBP (mm Hg)	71.66 (10.84)	70.37 (11.88)	71.01 (11.40)	**0.006** ^a^*
TG (mg/dl)	112.00 (84.00-164.00)	103.00 (79.00-153.00)	109.00 (81.00-159.00)	**0.001** ^c^*
FPG (mg/dl)	102.80 (29.96)	105.46 (36.32)	104.15 (33.36)	0.479 ^c^
HDL (mg/dl)	40.00 (35.00-46.00)	45.00 (39.00-51.00)	43.00 (37.00-49.00)	**<0.001** ^c^*
High FPG (%)	413 (36.4)	447 (38.1)	860 (37.3)	0.393^b^
High WC (%)	91 (8)	541 (46.1)	632 (27.4)	**<0.001^b^** *
High serum TG (%)	349 (30.7)	319 (27.2)	668 (28.9)	0.060 ^b^
Lows serum HDL (%)	540 (47.6)	823 (70.2)	945 (40.9)	**<0.001** ^b^*
High SBP (%)	158 (13.9)	199 (17)	357 (15.5)	**0.043** ^b^*
High DBP (%)	125 (11)	133 (11.3)	258 (11.2)	0.804 ^b^
Prevalence of Mets (%)	201 (17.7)	391 (33.3)	592 (25.6)	**<0.001** ^b^*

Data are shown as mean (standard deviation) or median (interquartile range) for normally and non-normally distributed continuous variables. Categorical variables are shown as numbers (percent).

^a^t-test Student, ^b^χ2 test, ^c^Mann-Whitney test.

*Statistically significant and shown in Bold Font.

BMI, body mass index; CRP, c-reactive protein; FPG, fasting plasma glucose; WC, waist circumference; TG, triglycerides; HDL, high-density lipoprotein cholesterol; SBP, systolic blood pressure; DBP, diastolic blood pressure; PA, Physical activity; MET, metabolic equivalent; HbA1c, Hemoglobin A1C; Cr, Creatinine.

### The number of latent classes

The number of latent classes, best fitted in the study population, was determined using the BIC value and LMR LR test p-value in each sex separately. In both sexes, the BIC value decreased by adding each class to the previous one until class 3. By adding class 4, the BIC value started increasing again in both sexes. LMR LR test p-values were also significant in class 3 in both men and women. According to the indicators and test results, the model with 3 classes had a better fit than other models for both sexes. The fit indices of the model are summarized in **Table 2**.

**Table 2:** Model fit indices for latent class models of metabolic syndrome.

Number of subclasses	Number of parameters	df	AIC	BIC	Entropy	LMR LR test p-value	Class counts
Men							
1- class	6	57	6808.784	6838.991	—	—	1135
2- class	13	50	6574.923	6640.370	0.738	< 0.0001	171, 964
3- class^*^	**20**	**43**	**6500.833**	**6601.520**	**0.649**	**0.0006**	**132, 783, 220**
4- class	27	36	6497.418	6633.347	0.689	0.0474	56, 744, 106, 229
5- class	34	29	6500.564	6671.733	0.776	0.445	495, 472, 39, 48, 81
Women							
1- class	6	57	7890.487	7920.891	—	—	1173
2- class	13	50	7356.736	7422.611	0.721	< 0.0001	297, 876
3- class^*^	**20**	**43**	**7287.461**	**7388.807**	**0.625**	**< 0.0001**	**228, 696, 249**
4- class	27	36	7278.420	7415.238	0.650	0.0266	124, 105, 695, 249
5- class	34	29	7281.711	7453.999	0.681	0.1882	70, 217, 144, 46, 696

In this research, the model selection criterion was based on the lower BIC values and significant LMR LR test p-value.

AIC, Akaike Information Criterion; BIC, Bayesian Information Criterion; LMR, Lo-Mendell-Rubin likelihood ratio test.

*The optimal class number according to the model fit criteria and also shown in bold font.

### Latent class profiles

**Figure 2:**
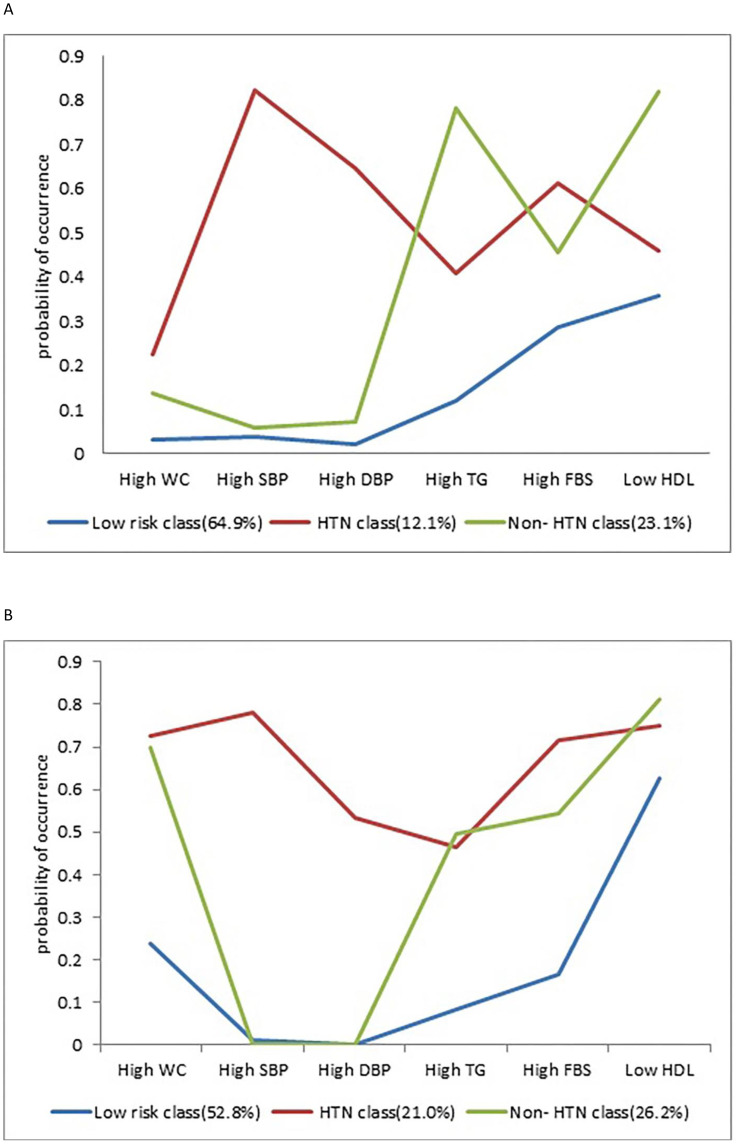
Probabilities of metabolic syndrome items in the 3 latent classes **(A)** Men, **(B)** Women.

All participants were assigned to the class where they were most likely to have similar characteristics. One of the classes was named “metabolic syndrome with Hypertension (HTN)”, with a prevalence of 11.6% in men and 19.4% in women, showing a high probability for at least 4 components of metabolic syndrome, especially high SBP (men: 82.4%, women: 78.1%). The other class was called “ metabolic syndrome without HTN (Non-HTN)” which had a prevalence of 19.4% in men and 21.2% in women. In this class, at least 3 components of metabolic syndrome had high probability but high SBP and high DBP were not among those components. The two major prevalent components of metabolic syndrome in this group were high TG and low HDL in men and High WC and low HDL in women. In the “Low Risk” class, which included 68.9% of men and 59.3% of women, the probability of all components of metabolic syndrome were low except for low HDL in women. (**Table 3**) (**Figure 2**).

**Table 3:** The prevalence of latent classes and the estimated probability of observed metabolic syndrome components for each class type.

Classes type	Low risk class	Mets with HTN class	Mets without HTN class
Men			
N (Latent class prevalence)	783 (0.689%)	132 (0.116%)	220 (0.194%)
High WC	0.033 (0.011,0.054)	0.224 (0.139,0.314)	0.138 (0.048,0.228)
High SBP	0.040 (0.012,0.067)	**0.824** (0.618,1.029)	0.059 (0.139,0.314)
High DBP	0.022 (0.001,0.043)	**0.645** (0.470,0.838)	0.074 (0.015,0.133)
High TG	0.120 (0.005,0.245)	0.408 (0.282,0.533)	0.**783** (0.479,1.086)
High FBS	0.285 (0.239,0.330)	**0.611** (0.509,0.713)	0.458 (0.358,0.558)
Low HDL	0.357 (0.276,0.437)	**0.460** (0.155,0.615)	0.**818** (0.557,1.078)
Women			
N (Latent class prevalence)	696 (0.593%)	228 (0.194%)	249 (0.212%)
High WC	0.238 (0.136,0.339)	**0.725** (0.662,0.787)	**0.699** (0.529,0.869)
High SBP	0.011 (0.005,0.027)	**0.781** (0.704,0.857)	0.000 (0.000,0.000)
High DBP	0.002 (0.007,0.012)	**0.535** (0.462,0.607)	0.000 (0.000,0.000)
High TG	0.084 (0.011,0.156)	0.464 (0.393,0.534)	0.497 (0.307,0.687)
High FBS	0.168 (0.095,0.240)	**0.715** (0.648,0.782)	**0.544** (0.381,0.706)
Low HDL	**0.627** (0.566,0.688)	**0.751** (0.692,0.810)	**0.812** (0.717,0.906)

For metabolic syndrome components data are presented as probability (95% Confidence Interval).

The probability of a “No” response can be calculated by subtracting the item-response probabilities shown above from 1.

Item-response probabilities >.5 in bold to facilitate interpretation.

FPG, fasting plasma glucose; WC, waist circumference; TG, triglycerides; HDL, high-density lipoprotein cholesterol; SBP, systolic blood pressure; DBP, diastolic blood pressure; Mets, metabolic syndrome; HTN, hypertension.

**Table 4** presents the characteristics of participants in each latent class of metabolic syndrome. In Men, age was significantly different between all 3 classes. HTN class was the oldest and the low-risk class was the youngest. BMI was not significantly different between the two classes of metabolic syndrome but the low-risk group had a significantly lower BMI compared to the other classes. Men in the HTN class also had a significantly higher incidence of diabetes and were simultaneously more physically active. Mean WC, SBP, DBP, FPG, and serum TG were significantly different between all the 3 classes. HTN class had the highest mean SBP, DBP, FPG, and WC and Non-HTN class had the highest mean serum TG. Serum HDL was significantly lower only in the non-HTN class compared to the other two classes. In comparison to the low-risk class, both the HTN and Non-HTN classes showed significantly higher levels of HbA1c, serum CRP, and Cr. Nevertheless, the difference between the two groups was not considered statistically significant. Serum vitamin D levels were not significantly different among the 3 classes in men.

**Table 4:** Characteristics of participants by latent classes of metabolic syndrome.

	Low-Risk class	HTN class	Non-HTN class	P value
Men (n=1135)				
Age (year)	42.40 (12.53)	50.64 (11.74)	44.57 (10.27)	**<0.001** ^a^ *
BMI (kg/m^2^)	25.55 (3.93)	29.17 (4.24)	28.48 (4.02)	**<0.001** ^a^ *
Vitamin D (ng/mL)	19.60 (14.10-24.61)	19.57 (13.22-24.12)	18.60 (14.22-23.17)	0.344 ^b^
CRP (mg/L)	1.00 (0.27-2.20)	1.20 (1.00-3.00)	1.60 (0.90-3.27)	**<0.001** ^b*^
Diabetes (%)	46 (5.9)	29 (22.0)	30 (13.6)	<0.001 ^c^*
Positive Smoking status, n (%)	183 (23.4)	28 (21.2)	54 (24.5)	0.77 **^c^**
PA (MET)	4126.0 (2655.0-5407.0)	4483.5 (3053.5-5881.2)	4050.5 (2722.5-5265.0)	**0.024** ^b*^
HbA1c (%)	4.56 (4.00-4.60)	4.56 (4.32-5.07)	4.56 (4.10-4.90)	<0.001 ^b^*
Cr (mg/dl)	1.12 (1.04-1.24)	1.18 (1.06-1.30)	1.16 (1.08-1.25)	0.001 ^b^*
WC (cm)	86.09 (9.58)	95.77 (10.34)	93.13 (9.25)	**<0.001** ^a^ *
SBP (mm Hg)	109.00 (100.00-117.50)	137.50 (130.00-146.87)	115.00 (107.50-120.00)	**<0.001** ^b^ *
DBP (mm Hg)	68.35 (9.08)	87.67 (9.38)	73.86 (7.94)	**<0.001** ^a^ *
TG (mg/dl)	98.00 (78.00-126.00)	129.50 (93.25-191.25)	200.00 (169.25-271.25)	**<0.001** ^b^ *
FPG (mg/dl)	94.00 (88.00-100.00)	104.50 (98.00-120.00)	98.00 (91.00-106.00)	**<0.001** ^b^ *
HDL (mg/dl)	43.00 (37.00-48.00)	42.00 (35.00-48.00)	34.00 (31.00-37.00)	**<0.001** ^b^ *
Women (n=1173)				
Age (year)	37.32 (11.39)	52.45 (7.98)	48.22 (9.61)	**<0.001** ^a^ *
BMI (kg/m^2^)	27.69 (5.39)	33.13 (5.63)	32.12 (4.21)	**<0.001** ^a^ *
Vitamin D (ng/mL)	13.50 (8.21-20.54)	16.25 (9.65-24.07)	16.10 (9.56-25.30)	**<0.001** ^b^ *
CRP (mg/L)	1.60 (0.62-3.90)	3.05 (1.00-6.72)	3.10 (1.00-6.00)	**<0.001** ^b^ *
Diabetes (%)	34 (4.9)	83 (36.4)	83 (33.3)	<0.001 ^c^*
Positive Smoking status, n (%)	3 (0.4)	0 (0.0)	2 (0.8)	**0.40 ^c^**
PA (MET)	3388.0 (2308.7-4780.0)	3635.0 (2297.5-5327.5)	2893.0 (1997.0-4185.5)	**<0.001** ^b^ *
HbA1c (%)	4.56 (4.00-4.56)	4.60 (4.50-5.50)	4.56 (4.568-4.90)	**<0.001** ^b^ *
Cr (mg/dl)	0.95 (0.87-1.03)	0.97 (0.88-1.06)	0.96 (0.88-1.05)	**0.028** ^b^ *
WC (cm)	82.26 (10.89)	94.93 (10.87)	95.87 (9.59)	**<0.001** ^a^ *
SBP (mm Hg)	101.25 (95.00-110.00)	137.50 (130.37-147.50)	110.00 (102.50-119.00)	**<0.001** ^b^ *
DBP (mm Hg)	65.84 (8.88)	85.91 (10.37)	68.78 (8.13)	**<0.001** ^a^ *
TG (mg/dl)	89.00 (69.00-113.00)	141.00 (100.00-187.00)	165.00 (113.00-213.50)	**<0.001** ^b^ *
FPG (mg/dl)	90.00 (85.00-96.00)	109.00 (97.00-134.75)	105.00 (100.00-123.50)	**<0.001** ^b^ *
HDL (mg/dl)	46.42 (11.92)	45.10 (10.40)	43.84 (9.13)	**0.005** ^a^ *

^a^ANOVA Test, ^b^Kruskal Wallis test, ^c^χ2 test.

Data are shown as mean (standard deviation) or median (interquartile range) for normally and non-normally distributed continuous variables. Categorical variables are shown as numbers (percent).

*P<0.05 was considered statistically significant and shown in Bold Font.

BMI, body mass index; FPG, fasting plasma glucose; WC, waist circumference; TG, triglycerides; HDL, high-density lipoprotein cholesterol; SBP, systolic blood pressure; DBP, diastolic blood pressure; CRP, c-reactive protein; HbA1c, Hemoglobin A1C; Cr, Creatinine; PA, Physical activity; MET, metabolic equivalent.

*Post Hoc* Tests: In the ANOVA Test, Bonferroni *post hoc* tests and in the Kruskal Wallis test adjusted Bonferroni *post hoc* tests were used for pairwise comparisons.

In Men: Significant mean difference between HTN and low-risk classes: Age, BMI, WC, DBP, SBP, TG, CRP, FPG, Cr, HbA1c, and PA.

Significant mean difference between HTN and non-HTN classes: Age, WC, DBP, SBP, TG, HDL, and FPG.

Significant mean difference between low-risk and non-HTN classes: BMI, WC, FPG, HDL, Age, DBP, SBP, CRP, TG, Cr, and HbA1c.

In Women: Significant mean difference between HTN and low-risk classes: Age, DBP, SBP, TG, BMI, Vitamin D, FPG, CRP, WC, Cr, and HbA1c.

Significant mean difference between classes HTN and non-HTN class: BMI, Age, DBP, SBP, TG, and PA.

Significant mean difference between classes low risk and non-HTN classes: BMI, Vitamin D, FPG, CRP and WC, HDL, Age, DBP, SBP, TG, HbA1c, Cr, and PA.

In women mean age and BMI were significantly different between all 3 classes. HTN class was the oldest and had the highest BMI while the low-risk class was the youngest with the lowest BMI. Women in the HTN group also had a significantly higher incidence of diabetes and were simultaneously more physically active. Mean serum SBP, DBP, and TG were significantly different between all three classes with the HTN class having the highest SBP and DBP and Non-HTN class having the highest mean serum TG level. The mean serum HDL level was significantly lower in the non-HTN class compared to the other classes. In comparison to the low-risk class, both the HTN and Non-HTN classes showed significantly higher levels of FPG, WC, HbA1c, serum vitamin D, CRP, and Cr. Nevertheless, the difference between the two groups was not considered statistically significant. (**Table 4**).

### Predictors of the metabolic syndrome classes

In men, using the “Low Risk” class as the reference class, BMI significantly predicted both classes of Mets (OR:1.26, CI:1.19-1.32, and OR:1.99, CI:1.15-1.24, for HTN and non-HTN classes respectively), while age only significantly predicted the HTN class. HbA1c (OR:1.42, CI:1.19-1.70, and OR:1.31, CI:1.12-1.54) and serum Cr (OR:2.93, CI:1.06-8.09, and OR:2.50, CI:1.06-5.88) were significant predictors of both HTN and non-HTN classes respectively, while serum vitamin D and CRP level were no significant predictors of metabolic syndrome classes.

In women, age (OR:1.16, CI:1.130-1.183, and OR:1.08, CI:1.065-1.103), BMI (OR:1.22, CI:1.171-1.268, OR:1.16, CI:1.124-1.207), HbA1c (OR:1.91, CI:1.60-2.29 and OR:1.75, CI:1.47-2.09), and serum Cr (OR:4.61, CI:1.72-12.34, and OR:3.23, CI:1.22-8.55) were significant predictors of both HTN and non-HTN Mets classes respectively, while serum CRP level only significantly predicted the HTN class (OR:1.03, CI:1.004-1.067). Serum vitamin D was not a significant predictor of metabolic syndrome classes (**Table 5**).

**Table 5:** Predictors of the Mets classes.

	HTN class OR (95% CI)	non-HTN class OR (95% CI)
Men (n=1135)		
Age (year)	**1.07 (1.047, 1.090)**	1.01 (0.999, 1.026)
BMI (kg/m^2^)	**1.26 (1.198, 1.329)**	**1.99 (1.150, 1.247)**
Serum Vitamin D (ng/mL)	0.99 (0.969, 1.006)	0.99 (0.982, 1.009)
Serum CRP (mg/L)	0.80 (1.004, 0.970)	1.01 (0.980, 1.034)
HbA1c (%)	**1.42 (1.19-1.70)**	**1.31 (1.12-1.54)**
Serum Cr (mg/dl)	**2.93 (1.06-8.09)**	**2.50 (1.06- 5.88)**
Women (n=1173)		
Age (year)	**1.16 (1.130, 1.183)**	**1.08 (1.065, 1.103)**
BMI (kg/m^2^)	**1.22 (1.171, 1.268)**	**1.16 (1.124, 1.207)**
Serum Vitamin D (ng/mL)	0.99 (0.977, 1.001)	1.00 (0.994, 1.013)
Serum CRP (mg/L)	**1.03 (1.004, 1.067)**	1.03 (0.977, 1.056)
HbA1c (%)	**1.91 (1.60-2.29)**	**1.75 (1.47-2.09)**
Serum Cr (mg/dl)	**4.61 (1.72-12.34)**	**3.23 (1.22-8.55)**

The reference category in classes is: low-risk class.

Data are shown as Odds Ratio (OR) and 95% Confidence Interval (CI). Multinomial Regression was used to evaluate the association between subclasses of Mets and the risk factors where low risk class served as the reference group.

BMI, body mass index; CRP, c-reactive protein; HbA1c, Hemoglobin A1C; Cr, Creatinine. Bold Font: statistically significant predictors (P<0.05).

### Association between latent classes and NAFLD

Using the “Low Risk” class as the reference class, both classes of metabolic syndrome significantly increased the OR for NAFLD in both men and women. After combining data from both sexes, latent class analysis revealed the same pattern across the population. In women, both HTN and Non-HTN classes resulted in a considerably higher OR for NAFLD compared to men even after adjustments for other covariates (age, serum vitamin D, serum c-reactive protein, diabetes, physical activity, and smoking status) (HTN class OR: 4.20 vs 2.94; non-HTN class OR: 5.60 vs 3.12 in women and men respectively) (**Table 6**).

**Table 6:** Association between latent classes of metabolic syndrome and NAFLD.

Low-Risk class	Men		Women		Total	
	Single OR(CI)	Multiple OR(CI)	Single OR(CI)	multiple OR(CI)	Single OR(CI)	multiple OR(CI)
	Reference	Reference	Reference	Reference	Reference	Reference
HTN class	**3.14** **(2.13-4.64)**	**2.94** **(1.98-4.37)**	**5.52** **(3.94-7.74)**	**4.20** **(2.96-5.95)**	**4.29** **(3.33-5.52)**	**3.48** **(2.69- 4.51)**
Non-HTN class	**3.12** **(2.27-4.28)**	**3.17** **(2.30-4.35)**	**5.60** **(4.02-7.80)**	**4.68** **(3.35-6.59)**	**4.15** **(3.30-5.20)**	**3.74** **(2.97-4.71)**

Data are shown as Odds Ratio (OR) and 95% Confidence Interval (CI). Logistic regression was used to evaluate the association between subclasses of Mets and NAFLD where the low-risk class served as the reference group (adjusted for age, serum vitamin D, serum c-reactive protein, diabetes, physical activity, and smoking status).

*Statistically significant ORs are shown in Bold Font.

NAFLD, Non-alcoholic fatty liver; HTN, Hypertension.

## Discussion

Latent class analysis has proven to be instrumental in uncovering various patterns and subtypes of metabolic syndrome, thereby greatly enhancing our comprehension of the diversity within metabolic syndrome and its associated health consequences. Consequently, it serves as a valuable instrument for facilitating a more sophisticated comprehension of the condition and potentially guiding personalized treatment approaches.

In this particular study, the utilization of the latent class analysis allowed us to identify three distinct classes: HTN, non-HTN, and low-risk classes. The primary distinguishing factor of the HTN class was the presence of hypertension, indicated by high SBP or DBP. Additionally, hyperglycemia played a significant role in this class. On the other hand, the non-HTN class was characterized by dyslipidemia, specifically high serum TG and/or low HDL, with a very low likelihood of hypertension.

The occurrence of subclasses of metabolic syndrome in this investigation aligns with numerous prior investigations (17–20, 42, 43). Ghahramanloo et al. conducted a study in Qom, Iran, which identified three distinct latent classes among urban adult men (non-Mets, 55.1%; at risk, 21.3%; and Mets, 23.6%). Consistent with our findings in men, one of these classes exhibited a higher prevalence of high TG and low HDL, while the other class had a higher prevalence of hypertension. Additionally, the group with hypertension had a higher likelihood of hyperglycemia (20). Another study by the same author in Tehran, identified four latent classes of metabolic syndrome: non-Mets, low risk, high risk, and MetS. Similar to our findings, hypertension and hyperlipidemia were the primary components of the last two classes, but in contrast to our results, hyperglycemia was more closely associated with hyperlipidemia (17). In another study by Galvão et al, researchers used latent class analysis to identify different patterns of metabolic syndrome among women in the ELSA-Brasil cohort. Similar to our own findings, they identified three patterns that one them was characterized by high serum TG and low HDL, while the other pattern was associated with central obesity, hyperglycemia, and hypertension. The third pattern had low probabilities of all abnormalities, except for central obesity (44). Another study analyzed data from the Multi-Ethnic Study of Atherosclerosis (MESA) to identify patterns of metabolic syndrome components. This study also highlighted the importance of hypertension, low HDL, and hyperglycemia in defining different subgroups of metabolic syndrome (45).

The existence of a HTN latent subgroup within the population diagnosed with metabolic syndrome where hypertension and hyperglycemia are the two most important components can be elucidated through various mechanisms. Prior studies suggest that insulin resistance and endothelial dysfunction could link hypertension and hyperglycemia. Insulin resistance impairs the ability of insulin to promote glucose uptake in tissues, leading to hyperglycemia. Meanwhile Insulin has vasodilatory effects. When cells are resistant to insulin, this vasodilatory effect is diminished, contributing to increased vascular resistance and hypertension (46). High blood glucose levels can also damage the endothelium. This damage impairs the production of nitric oxide (NO), a molecule that helps blood vessels relax (47). Additionally, hyperglycemia can lead to the formation of advanced glycation end-products (AGEs), which further damage the endothelium and promote inflammation, contributing to hypertension (47, 48). Insulin resistance and hyperglycemia can also activate the sympathetic nervous system, which increases heart rate and constricts blood vessels, leading to higher blood pressure (49).

Furthermore, research suggests that the impact of insulin resistance on endothelial function may vary depending on gender. One potential explanation for this is the presence of hormonal disparities. Estrogen is known to have a protective effect on endothelial function, whereas testosterone’s relationship with endothelial function is more intricate (50). Another factor to consider is the inflammatory response. Insulin resistance often coincides with an inflammatory state, and the inflammatory reaction may differ between males and females. Some studies propose that males might be more vulnerable to the adverse effects of inflammation on endothelial function (51). This phenomenon could clarify why men in the present study displayed elevated systolic blood pressure and hyperglycemia even in the absence of abdominal obesity (low prevalence of increased waist circumference), unlike women. Boyko et al. also suggest that this connection between hypertension and hyperglycemia may not be influenced by central adiposity (as was noted in men in the present study) (15).

Impairment in renal function could potentially be the other link connecting hyperglycemia and hypertension. Previous studies have shown that prediabetes can worsen kidney function, while chronic kidney disease can exacerbate hypertension by affecting the body’s fluid and electrolyte balance (52, 53). In contrast, hypertension can further advance chronic kidney disease by putting stress on the kidneys, causing damage to blood vessels, and reducing their ability to effectively filter waste products (54, 55). In the current study, both HbA1c and serum Cr were found to be significant predictors of metabolic syndrome classes, particularly the HTN class. This finding strengthens the connection between kidney function impairment, hyperglycemia, and hypertension. Endothelial dysfunction caused in all the above mechanisms could be a key factor in the adverse effects of hypertension and hyperglycemia, even in the absence of diabetes, leading to a decline in function, vascular issues, and Alzheimer’s disease (56–58). Therefore, the presence of both high blood pressure and hyperglycemia, as seen in the HTN latent class identified in this study, highlights the urgent need to address these conditions to prevent organ damage and associated complications.

In the current study, the presence of non-HTN latent subgroup that was characterized by dyslipidemia, specifically high serum TG and/or low HDL, with a very low likelihood of hypertension, could be explained through mechanisms related to lipoprotein metabolism and genetic factors that do not necessarily involve hypertension such as hepatic overproduction of VLDL (59), impaired lipoprotein lipase activity or lipoprotein metabolism (60, 61). Understanding these mechanisms is crucial for developing targeted interventions for metabolic syndrome subclasses.

Serum CRP and vitamin D levels were not significant predictors of metabolic syndrome classes in the present study, except for serum CRP for HTN class in women. CRP is a protein produced by the liver in response to inflammation, primarily triggered by interleukin-6 (IL-6) and other inflammatory cytokines (62). Research suggests that adipose tissue can produce inflammatory cytokines, leading to increased levels of CRP (63–65). In the current research women exhibit significantly higher BMI, waist circumference, and double serum CRP levels, indicating that the association between serum CRP level and HTN class in women might indicate a heightened inflammatory state in women, which can contribute to the pathophysiology of hypertension (66). Studies have also shown that CRP levels are higher in individuals with specific components of metabolic syndrome, such as abdominal obesity, insulin resistance, and hypertension (67–72).

Regarding the association between serum vitamin D level and metabolic syndrome, different studies have reported inconsistent results. While some research has indicated a higher likelihood of developing metabolic syndrome and its components with low serum vitamin D levels (73, 74), others have not found such a connection (75, 76). These results imply that the association between serum vitamin D levels and metabolic syndrome may vary among different populations and age groups. In Iran, factors like limited sunlight exposure, low intake of vitamin D-rich foods (77), cultural norms regarding clothing that limits sun exposure (78), economic constraints affecting access to nutrition and healthcare (77), low physical activity, and air pollution (79, 80) all contribute to a high prevalence of vitamin D deficiency. The mean serum vitamin D level of 17.30 ng/mL in this study further underscores the prevalence of Vitamin D deficiency in Iran. This deficiency may complicate the ability to identify associations between vitamin D levels and metabolic syndrome classes, as low levels of serum vitamin D are commonly found in all classes, potentially masking differences that would otherwise be noticeable.

Understanding these factors can provide valuable context for interpreting the lack of association between vitamin D status and metabolic syndrome classes in our study, highlighting the complexity of this relationship within specific demographic groups.

Concerning the correlation between the identified metabolic syndrome classes and NAFLD, both the HTN and non-HTN classes exhibited a similar increase in the OR of NAFLD for each gender. However, the ORs of both classes were significantly higher in women compared to men, approximately three times higher. This notable difference in ORs can potentially be attributed to the higher prevalence of visceral adiposity in women within both metabolic syndrome classes. According to our finding 71.1% and 88.0% of women in HTN and Non-HTN classes had high waist circumference. In men, the frequencies were 35.2% and 38.5% for the HTN and Non-HTN classes, respectively (**Supplementary Table 1**). Visceral adiposity is a crucial risk factor for NAFLD, and its higher occurrence in women may explain the considerably higher ORs of both HTN and non-HTN classes for NAFLD in women compared to men. Our findings align with the study conducted by Ahanchi et al, which reported that the relationship between subclasses of metabolic syndrome and incident CVD varied by gender. They suggested that the etiology of metabolic syndrome involves multiple pathways, and it is necessary to reconsider the equal weighting of each component or the use of the same cut-off values in both genders (19).

Our findings indicate that there may be distinct latent classes within the defined metabolic syndrome that exhibit varying pathophysiology or different levels of risk for non-communicable diseases. The main finding in this study was the significance of hypertension in the classification of metabolic syndrome. Our study demonstrates a conditional probability of approximately 0 for SBP and DBP in both men and women in the “non-HTN” class, indicating excellent classification and high specificity of the SBP and DBP components for these classes. Conversely, there is a simultaneous high probability of hyperglycemia in the HTN class, suggesting a shared underlying mechanism for these two components, separate from dyslipidemia. Another noteworthy observation is the presence of elevated visceral adiposity in both classes among women, but not men, which may indicate a disparity in the role of visceral adiposity in the pathophysiological mechanism of metabolic syndrome between the two sexes.

Comprehension of the diversity within metabolic syndrome and its related health consequences helps in better identifying individuals who may be at a higher or lower risk of developing related conditions like NAFLD. Precise risk categorization allows for focused interventions and more frequent monitoring of those at high risk. Customizing interventions according to the specific metabolic syndrome subclass can maximize the effectiveness of lifestyle changes, medication, and monitoring methods.

The study’s notable strength lies in its utilization of a substantial population-based cohort study, thereby enhancing the credibility and reliability of our findings. Furthermore, we performed latent class analysis separately for each gender, thereby presenting distinct patterns for both males and females. This study also offers important information on the subclasses of metabolic syndrome and their connection to NAFLD. Nevertheless, it is crucial to recognize the limitations of this research. It is vital to acknowledge the potential impact of unobserved factors such as socioeconomic status, genetic traits, and lifestyle variables influencing the results. Being cross-sectional and observational is another limitation to consider when interpreting the results. Furthermore, although we have adjusted the association between latent classes of metabolic syndrome and NAFLD we suggest future-tailored research with higher sample sizes that can assess the associations in covariate-based stratified groups in each sex to address the unique health needs of both male and female participants based on the gender-specific insights from our study. Overcoming these limitations in future studies will improve our comprehension of the intricate relationships between NAFLD and metabolic well-being.

## Conclusion

In the northern region of Iran, the latent class analysis revealed the presence of three distinct classes of metabolic syndrome: HTN, Non-HTN, and low-risk classes. Hypertension played a crucial role in determining these classes. Furthermore, both HTN and Non-HTN classes exhibited a higher prevalence of visceral adiposity and served as stronger predictors of NAFLD in women. Notably, serum CRP and vitamin D levels did not emerge as significant predictors of metabolic syndrome latent classes, except for serum CRP in the HTN class among women.

## Data Availability

The raw data supporting the conclusions of this article will be made available by the authors, without undue reservation.
