# Supplementary material for: The crucial role of hypertension in determining latent classes of metabolic syndrome in northern Iran and predictive power of these classes in non-alcoholic fatty liver: a gender-based insight
Source: Front Endocrinol (Lausanne). 2025 Feb 28;16:1405833. doi: 10.3389/fendo.2025.1405833 (PMC11906334; doi:10.3389/fendo.2025.1405833)
Supplement: Supplementary file 1 [file Table1.docx]

Supplementary Table 1. Distribution of metabolic syndrome components by latent classes of Metabolic syndrome ^a^

|  | Low-Risk class  N (%) | HTN class  N (%) | Non-HTN class N (%) | P value |
| --- | --- | --- | --- | --- |
| Men (n=1135) | | |  |  |
| WC >102 cm | 3.1 | 24.2 | 15.9 | <0.001 ***** |
| SBP ≥130 mm Hg | 3.7 | 86.4 | 6.8 | <0.001 * |
| DBP ≥85 mm Hg | 1.8 | 71.2 | 7.7 | <0.001 * |
| TG ≥150 mg/dl | 10.70 | 37.90 | 97.70 | <0.001 * |
| FPG ≥100 mg/dl | 28.5 | 72.0 | 43.2 | <0.001 * |
| HDL < 40 mg/dl | 34.9 | 41.7 | 96.4 | <0.001 * |
| Women (n=1173) | | |  |  |
| WC >88 cm | 23.0 | 71.1 | 88.0 | <0.001 * |
| SBP ≥130 mm Hg | 0.1 | 86.8 | 0.0 | <0.001 * |
| DBP ≥85 mm Hg | 0.0 | 58.3 | 0.0 | <0.001 * |
| TG ≥150 mg/dl | 8.5 | 45.2 | 63.1 | <0.001 * |
| FPG ≥100 mg/dl | 14.1 | 69.7 | 76.3 | <0.001 * |
| HDL < 50 mg/dl | 66.5 | 75.4 | 75.5 | 0.004 * |

a: χ2 test. Data are shown as as numbers (percent).

* P<0.05 was considered statistically significant.

**Abbreviations:** FPG: fasting plasma glucose, WC: waist circumference, TG: triglycerides, HDL: high-density lipoprotein cholesterol, SBP: systolic blood pressure, DBP: diastolic blood pressure, CRP: c-reactive protein.
